# Membrane-bound and soluble Fas ligands have opposite functions in photoreceptor cell death following separation from the retinal pigment epithelium

**DOI:** 10.1038/cddis.2015.334

**Published:** 2015-11-19

**Authors:** H Matsumoto, Y Murakami, K Kataoka, S Notomi, D Mantopoulos, G Trichonas, J W Miller, M S Gregory, B R Ksander, A Marshak-Rothstein, D G Vavvas

**Affiliations:** 1 Angiogenesis Laboratory, Department of Ophthalmology, Massachusetts Eye and Ear Infirmary, Harvard Medical School, Boston, MA, USA; 2 Department of Ophthalmology, Massachusetts Eye and Ear Infirmary, Harvard Medical School, Schepens Eye Research Institute, Boston, MA, USA; 3 Department of Medicine, University of Massachusetts Medical School, Worcester, MA, USA

## Abstract

Fas ligand (FasL) triggers apoptosis of Fas-positive cells, and previous reports described FasL-induced cell death of Fas-positive photoreceptors following a retinal detachment. However, as FasL exists in membrane-bound (mFasL) and soluble (sFasL) forms, and is expressed on resident microglia and infiltrating monocyte/macrophages, the current study examined the relative contribution of mFasL and sFasL to photoreceptor cell death after induction of experimental retinal detachment in wild-type, knockout (*FasL*−/−), and mFasL-only knock-in (ΔCS) mice. Retinal detachment in *FasL*−/− mice resulted in a significant reduction of photoreceptor cell death. In contrast, ΔCS mice displayed significantly more apoptotic photoreceptor cell death. Photoreceptor loss in ΔCS mice was inhibited by a subretinal injection of recombinant sFasL. Thus, Fas/FasL-triggered cell death accounts for a significant amount of photoreceptor cell loss following the retinal detachment. The function of FasL was dependent upon the form of FasL expressed: mFasL triggered photoreceptor cell death, whereas sFasL protected the retina, indicating that enzyme-mediated cleavage of FasL determines, in part, the extent of vision loss following the retinal detachment. Moreover, it also indicates that treatment with sFasL could significantly reduce photoreceptor cell loss in patients with retinal detachment.

Separation of photoreceptors from underlying retinal pigment epithelium (RPE), as seen rhegmatogenous retinal detachment,^1^ causes photoreceptor cell death, resulting in permanent vision loss. In a majority of cases, photoreceptor cell death occurs even if the retina is successfully reattached surgically. Separation of photoreceptors from the RPE also contributes to photoreceptor cell death in age-related macular degeneration,^2^ diabetic retinopathy,^3^ and retinopathy of prematurity.^4^ Therefore, it is important to define the mechanism(s) of photoreceptor cell death in the detached retina and establish therapeutic targets that prevent photoreceptor loss and the subsequent decrease in visual acuity.

Fas ligand (FasL) exists as a trimer in the cell membrane, whereas the Fas receptor (FasR or Fas) is expressed as a monomer. When FasL-positive cells come in contact with Fas-positive cells, Fas/FasL binding causes trimerization of Fas receptors that signals the binding of Fas-associated death domain (FADD) adaptor proteins; this triggers a sequential signaling cascade that recruits and activates caspase 8, caspase 3, and finally caspase-activated DNAse (CAD) that ultimately enters the nucleus and cleaves DNA, resulting in apoptotic cell death.^5, 6, 7^ Although Fas signaling is mainly associated with this apoptotic cell death pathway, it has also been reported that, when FasL triggers Fas receptors in cells that inhibit or lack caspase 8, an alternative death pathway is activated that is mediated by receptor interacting protein (RIP) kinase, leading to necrotic cell death.^8, 9, 10, 11^ Thus, Fas signaling can induce not only apoptosis but also necrosis. It is important to understand whether photoreceptors die via apoptosis or necrosis following the retinal detachment, as necrotic cell death typically causes infiltration of inflammatory cells that may cause bystander death of surrounding normal cells, increasing loss of photoreceptors.

FasL is a type II transmembrane protein in the TNF family, and like many genes in this group, FasL exists in several different forms.^12^ The membrane-bound form (mFasL) can be cleaved from the cell surface by metalloproteinases to produce a truncated soluble product (sFasL) derived from the extracellular domain.^13^ Prior studies demonstrated that apoptosis triggered by FasL requires extensive oligomerization of the Fas receptor to activate the death-inducing signaling complex (DISC).^14^ Although both mFasL and sFasL contain the trimerization domain and can bind the Fas receptor, the naturally cleaved form of sFasL is unable to oligomerize the Fas receptor and trigger apoptosis.^14, 15, 16^ For this reason, mFasL but not sFasL induces apoptosis in Fas-positive cells. In addition, reports indicate that sFasL *blocks* mFasL-mediated apoptosis via steric hindrance when sFasL binds the Fas receptor and physically blocks the binding of mFasL and oligomerization of the Fas receptor.^17, 18^

Within the eye, Fas is expressed widely on cells in the anterior and posterior segment, whereas FasL has very limited expression and is found only on corneal epithelial cells, microglia, astrocytes, and RPE cells. The constitutive expression of FasL on corneal epithelial cells and RPE cells is necessary to maintain ocular immune privilege by inducing apoptosis of infiltrating Fas-positive inflammatory cells, which limits inflammation and subsequent tissue damage of ocular tissues.^19^ Although FasL limits inflammation, other reports indicate that mFasL promotes inflammation, and that sFasL is non-inflammatory or blocks mFasL-triggered inflammation. Therefore, the overall function of FasL is the result of the separate contributions of mFasL and sFasL, which have opposing functions in apoptosis and inflammation.^17, 18^

The function of Fas/FasL in photoreceptor death was examined by our group in a rat model of retinal detachment,^5^ as well as other groups who observed a significant decrease in photoreceptor apoptosis in FasL^*gld*^ and Fas^*lpr*^ mutant mice.^6, 20^ However, although these data demonstrate clearly that this pathway contributes to photoreceptor cell loss in detached retinas, these studies did not examine the contribution of the different forms of FasL (mFasL and sFasL). Moreover, these previous studies used FasL^*gld*^ and Fas^*lpr*^ mutant mice, which have specific point mutations in FasL and Fas (gld and lpr mutations, respectively) that reduce but do not block completely Fas/FasL signaling;^21^ thus, the overall contribution of FasL in photoreceptor cell death is not completely known.

In our current study, we examined the overall contribution of FasL using FasL-knockout (*FasL*−/−) mice and the relative contribution of mFasL and sFasL in the death of photoreceptors following experimentally induced retinal detachment. Fas/FasL signaling was completely eliminated in *FasL*−/− mice and sFasL was eliminated in ΔCS mice that possess an exchange knock-in mutation in the FasL metalloproteinase cleavage site, producing mice that express increased levels of mFasL and no sFasL.^22^ The potential neuroprotective effects of sFasL in photoreceptor cell death were also examined during retinal detachment.

## Results

### FasL mediates photoreceptor cell death induced by retinal detachment

**Figure 1:**
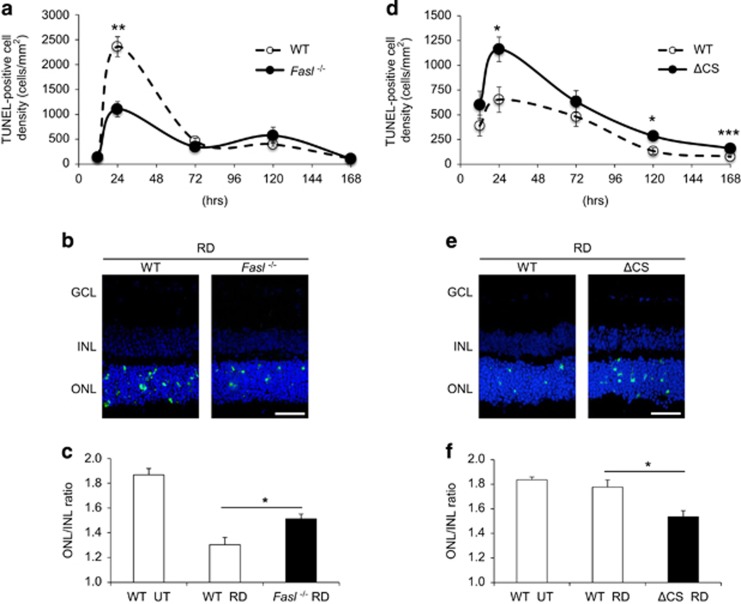
FasL mediates photoreceptor cell death induced by retinal detachment. (**a**) Time course of TUNEL-positive cell density in outer nuclear layer (ONL) in BALB/c WT and BALB/c *FasL*−/− mice (*n*=6 each group and time point). The peak of TUNEL-positive cell density was day 1 in both groups. BALB/c *FasL*−/− mice showed significantly less photoreceptor cell death than BALB/c WT mice 1 day after retinal detachments (**P<0.01). (**b**) TUNEL (green) and TO-PRO-3 (blue) staining at 24 h after retinal detachment. Only ONL showed TUNEL-positive cells in both BALB/c WT and BALB/c *FasL*−/− mice. (**c**) ONL/INL (inner nuclear layer) ratio 7 days after retinal detachment (*n*=6 each). ONL/INL ratio was significantly higher in BALB/c *FasL*−/− than in BALB/c WT mice (*P<0.05). (**d**) Time course of TUNEL-positive cell density in ONL in B6129 WT and B6129 ΔCS mice (*n*=6 each group and time point). The peak of TUNEL-positive cell density was on day 1 in both groups. B6129 ΔCS mice showed significantly more photoreceptor cell death than B6129 WT mice at 24 h (*P<0.05), 120 h (*P<0.05), and 168 h (*P<0.001) after retinal detachment. (**e**) TUNEL (green) and TO-PRO-3 (blue) staining at 24 h after retinal detachment. Only ONL showed TUNEL-positive cells in both B6129 WT and B6129 ΔCS mice. (**f**) ONL/INL ratio 7 days after retinal detachment (*n*=6 each). ONL/INL ratio was significantly lower in B6129 ΔCS than in B6129 WT mice (*P<0.05). Scale bar, 50 *μ*m. The graphs show mean±S.E.M. GCL, ganglion cell layer; UT, untreated

To determine the relative contribution of FasL, mFasL, and sFasL in the death of photoreceptors following retinal detachment, we used two strains of mice that we previously identified as susceptible (BALB/c) and resistant (B6129SF2) to photoreceptor death following an induced retinal detachment.^23^ BALB/c *FasL*−/− mice were produced by an eight nucleotide deletion in the *FasL* gene that results in a splicing error and frameshift mutation (Supplementary Figure 1A and B). B6129 ΔCS mice were produced by an exchange mutation in *FasL* gene sequence, which replaces four residues bracketing two enzymatic cleavage sites (Supplementary Figure 1C). The retinas of *FasL*−/− and ΔCS mice were not significantly different from WT mice (Supplementary Figure 2). We first determined photoreceptor cell death over time after induction of retinal detachment by TdT-dUTP terminal nick-end labeling (TUNEL) assay in BALB/c WT and BALB/c *FasL*−/− mice. We previously demonstrated that the number of TUNEL-positive cells peaked on day 1.^23^ Therefore, we analyzed TUNEL-positive cell density in outer nuclear layer (ONL) at 0.5, 1, 3, 5, and 7 days after retinal detachment. The ONL/inner nuclear layer (INL) ratio was then assessed on day 7. A significant reduction in photoreceptor cell death was observed in BALB/c *FasL*−/− mice as compared with BALB/c WT mice at 24 h after retinal detachment (WT: 2359±202 cells/mm^2^ and *FasL*−/−: 1105±154 cells/mm^2^; **P<0.01; Figure 1a). As expected, the other retinal layers showed no TUNEL-positive cells at any time point (Figure 1b). Furthermore, BALB/c *FasL*−/− mice displayed significantly higher ONL/INL ratios as compared with BALB/c WT mice 7 days after retinal detachment (WT: 1.30±0.06 and *FasL*−/−: 1.51±0.04; *P<0.05; Figure 1c), suggesting that photoreceptor cell death was prevented in FasL-deficient mice.

ΔCS mice have an exchange mutation in the FasL metalloproteinase cleavage site that prevents cleavage of mFasL, resulting in increased levels of mFasL and no sFasL.^22^ Therefore, we speculated that even in the resistant B6129SF2 mouse strain, the increased levels of mFasL and the lack of sFasL in the B6129 ΔCS mice would result in more photoreceptor death following retinal detachment as compared with B6129 WT mice. Consistent with our hypothesis, B6129 ΔCS mice displayed significantly increased photoreceptor cell death at 24 h (WT: 654±128 cells/mm^2^ and ΔCS: 1161±124 cells/mm^2^; *P<0.05), 120 h (WT: 134±33 cells/mm^2^ and ΔCS: 286±30 cells/mm^2^; *P<0.05), and 168 h (WT: 78±8 cells/mm^2^ and ΔCS: 160±11 cells/mm^2^; ***P<0.001) after retinal detachment (Figure 1d). The other retinal layers showed no TUNEL-positive cells at any time point (Figure 1e). Moreover, B6129 ΔCS mice displayed significantly lower ONL/INL ratio as compared with B6129 WT mice (WT: 1.78±0.06 and ΔCS: 1.53±0.05; *P<0.05; Figure1f). We conclude from these results that preventing enzymatic cleavage of FasL significantly increased photoreceptor cell death following retinal detachment.

### FasL mediates both photoreceptor apoptosis and necrosis after retinal detachment

**Figure 2:**
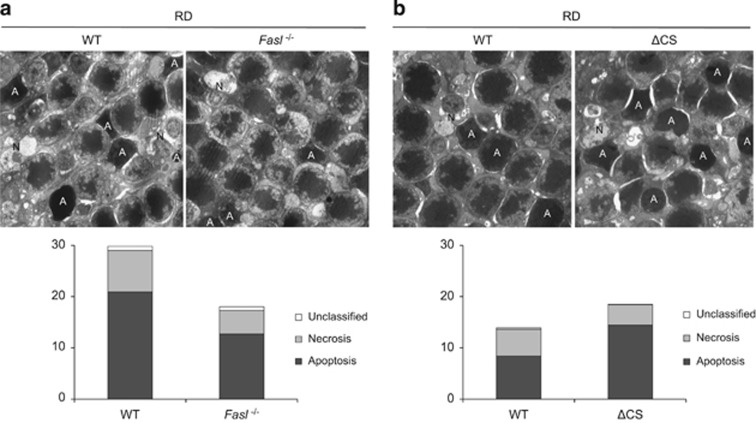
FasL mediates both photoreceptor apoptosis and necrosis after retinal detachment. (**a**) BALB/c *FasL*−/− mice showed significantly less apoptotic and necrotic photoreceptor cell deaths than BALB/c WT mice at 24 h after retinal detachment (*P*<0.05 each). (**b**) B6129 ΔCS mice exhibited significantly more apoptotic photoreceptor cell death than B6129 WT mice at 24 h after retinal detachment (*P*<0.05 each), whereas there was no significant difference in necrotic photoreceptor cell death between them. A, apoptosis; N, necrosis

To further investigate the type of photoreceptor cell death, we performed transmission electron microscopy (TEM) on the detached retina at 24 h after detachment (Figure 2a). BALB/c *FasL*−/− mice showed significantly less apoptotic photoreceptor cell death than BALB/c WT mice (WT: 21.0±1.8% and *FasL*−/−: 12.7±2.0% *P<0.05). Interestingly, necrotic photoreceptor cell death was also significantly reduced in *FasL*−/− mice (WT: 8.1±1.1% and *FasL*−/−: 4.6±0.3% *P<0.05). These results indicate that abolishing FasL function in susceptible BALB/c mice can rescue both apoptotic and necrotic photoreceptor death following retinal detachment. On the other hand, in B6129SF2-resistant mice, ΔCS mice displayed significantly more apoptotic photoreceptor cell death than WT mice (WT: 8.4±0.9% and ΔCS: 14.4±1.5% *P<0.05). However, there was no significant difference in necrotic photoreceptor cell death between the two groups (WT: 5.2±0.9% and ΔCS: 4.0±0.4%). These results indicate that preventing enzymatic cleavage of FasL accelerated apoptotic (but not necrotic) photoreceptor cell death.

### Retinal detachment-induced inflammatory cytokine expression is reduced in *FasL*−/− mice and increased in ΔCS mice

**Figure 3:**
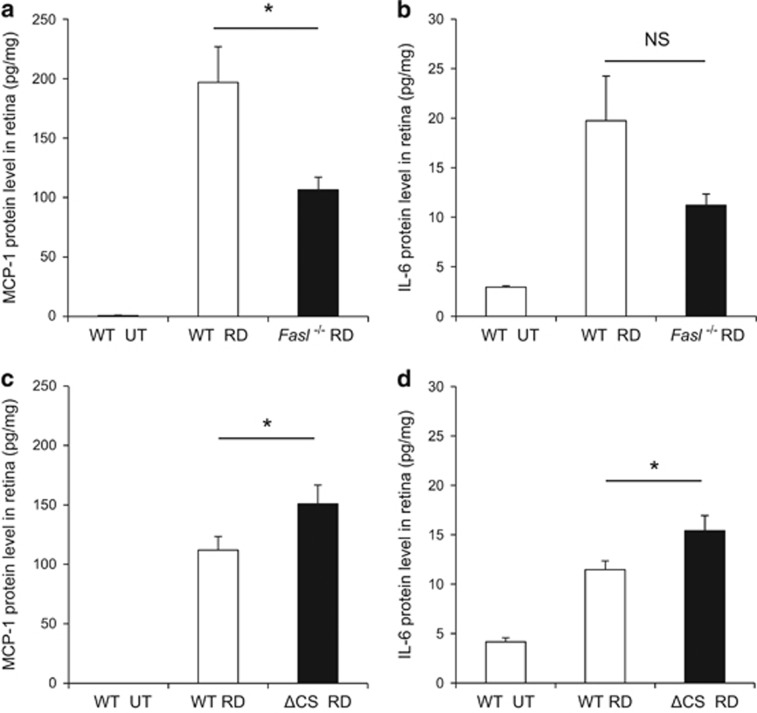
Retinal detachment-induced inflammatory cytokine expression is reduced in *FasL*−/− mice but increased in ΔCS mice. (**a** and **b**) ELISA to detect MCP-1 (**a**) and IL-6 (**b**) in the BALB/c WT and BALB/c *FasL*−/− retinas at 24 h after retinal detachment (*n*=8 each). MCP-1 generation was significantly suppressed in BALB/c *FasL*−/− mice (*P<0.05). IL-6 expression was reduced (but not significant) in BALB/c *FasL*−/− than in BALB/c WT mice. (**c** and **d**) ELISA to detect MCP-1 (**c**) and IL-6 (**d**) in the B6129 WT and B6129 ΔCS retinas at 24 h after retinal detachment (*n*=8 each). MCP-1 and IL-6 generation was significantly increased in B6129 ΔCS than in B6129 WT mice (*P<0.05)

We previously demonstrated that monocyte chemoattractant protein 1 (MCP-1) is an essential mediator of early infiltration of macrophage/microglia after retinal detachment.^24^ Zacks *et al.*^25, 26^ observed that interleukin 6 (IL-6) was also increased in the retina after retinal detachment using gene microarray analysis. IL-6 is one of the stress-response genes related to inflammation as well as hematopoiesis, angiogenesis, cell differentiation, and neuronal survival, which can be produced by retinal cells other than macrophages. We evaluated MCP-1 and IL-6 expression levels by ELISA at 24 h after retinal detachment in the whole retina. In susceptible BALB/c mice, MCP-1 was significantly lower in *FasL*−/− as compared with WT mice (Figure 3a). However, there was no significant difference in IL-6 (Figure 3b). In contrast, in resistant B6129SF2 mice, ΔCS mice showed a significant increase in both MCP-1 and IL-6 levels compared with WT mice (Figures 3c and d). These data indicate FasL triggers an increase in pro-inflammatory MCP-1 and IL-6, which is further increased when enzymatic cleavage of FasL is blocked.

### Inflammatory cell infiltration after retinal detachment is accelerated in *FasL*−/− mice

We next analyzed the time course of migrating CD11b+ macrophage/microglial cells following retinal detachment. Interestingly, despite the lower MCP-1 levels observed in the absence of FasL, the infiltration of CD11b-positive cells was accelerated, resulting in a significant increase in the number of infiltrating CD11b-positive cells in *FasL*−/− compared with WT mice 24 h after retinal detachment (Supplementary Figure S3A and B). By 72 h, the number of CD11b-positive cells was equal in both *FasL*−/− and WT mice. On the other hand, although ΔCS mice showed significantly higher MCP-1 and IL-6 levels compared with WT mice, the number of infiltrating CD11b-positive cells was not significantly different at all time points examined (Supplementary Figure S3C and D).

### Differential M1 and M2 subtype of subretinal macrophages after induction of retinal detachment

**Figure 4:**
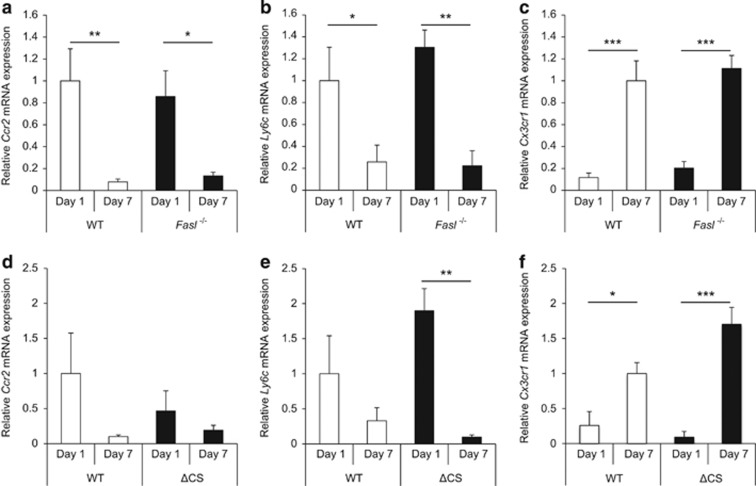
Classically and alternatively activated subretinal macrophages after induction of retinal detachment. (**a**–**c**) *Ccr2*, *Ly6c*, and *Cx3cr1* mRNAs expression in the subretinal space was evaluated by LCM followed by qPCR using BALB/c WT and BALB/c *FasL*−/− mice at 1 and 7 days after retinal detachment (*n*=6 each group and time point). *Ccr2* mRNA was significantly higher in day 1 than in day 7 in each group (WT: **P<0.01, *FasL*−/−: *P<0.05) (**a**). *Ly6c* mRNA was significantly higher in day 1 as compared with day 7 (WT: *P<0.05, *FasL*−/−: **P<0.01) (**b**). On the other hand, *Cx3cr1* mRNA was significantly higher in day 7 than in day 1 (WT: ***P<0.001, *FasL*−/−: ***P<0.001) (**c**). There were no significant differences in the three mRNAs expression between BALB/c WT and BALB/c *FasL*−/− mice. (**d**–**f**) *Ccr2*, *Ly6c*, and *Cx3cr1* mRNAs expression in the subretinal space in B6129 WT and B6129 ΔCS mice at 1 and 7 days after retinal detachment (*n*=6 each group and time point). *Ccr2* mRNA tended to be higher in day 1 than in day 7 in each group; however, the differences did not achieve statistical significance (**d**). *Ly6c* mRNA was significantly higher in day 1 as compared with day 7 in B6129 ΔCS mice (**P<0.01). B6129 WT mice showed higher *Ly6c* mRNA in day 1 than in day 7; however, the difference did not reach statistical significance (**e**). *Cx3cr1* mRNA was significantly higher in day 7 than in day 1 (WT: *P<0.05, ΔCS: ***P<0.001) (**f**). There were no significant differences in the three mRNAs expression between B6129 WT and B6129 ΔCS mice. The graphs show mean±S.E.M.

Classically and alternatively activated macrophages (M1 and M2, respectively) have been reported to display opposing functions during retinal degeneration.^27, 28^ Ccr2 and Ly6c are considered as M1 markers, while Cx3cr1 is a M2 marker.^27, 28, 29^ To determine the macrophage subtypes within the subretinal space following detachment of the retina, we evaluated *Ccr2*, *Ly6c*, and *Cx3cr1* mRNA expression in macrophage/microglia cells isolated on days 1 and 7 via laser capture microdissection (LCM) followed by quantitative real-time PCR (qPCR). In susceptible BALB/c mice, both WT and *FasL*−/− mice displayed significantly higher levels of *Ccr2* and *Ly6c* mRNA at day 1 as compared with day 7 (Figures 4a and b), while *Cx3cr1* mRNA was significantly higher at day 7 as compared with day 1 (Figure 4c). However, there were no significant differences in the expression of *Ccr2*, *Ly6c*, and *Cx3cr1* between WT and *FasL*−/− mice. Resistant B6129SF2 mice showed a similar pattern of expression, and there were no significant differences between ΔCS and WT mice (Figures 4d–f). Although the difference of some mRNA expressions between days 1 and 7 did not achieve statistical significance, there was an observable trend, and larger sample sizes might lead to statistical significance. These results indicate that, while the macrophages migrating into the subretinal space in this retinal detachment model are differentially activated depending on the time after detachment (with M1 seen in the early time points and M2 seen at later time points), the differences in subtypes were not affected by FasL.

### Soluble Fas ligand rescued photoreceptor cells from death after retinal detachment

**Figure 5:**
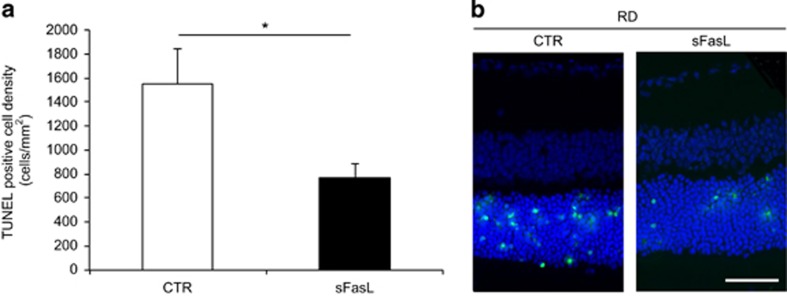
Subretinal injection of soluble Fas ligand attenuates photoreceptor cell death after retinal detachment. (**a**) TUNEL-positive cell density in the outer nuclear layer (ONL) in B6129 ΔCS mice at 24 h after retinal detachment. Recombinant murine soluble Fas ligand (sFasL) or control phosphate-buffered saline (CTR) was injected into the subretinal space when creating retinal detachment (n=12 each group). The eyes with sFasL showed significantly reduced photoreceptor cell death as compared with control eyes (*P<0.05). (**b**) TUNEL (green) and TO-PRO-3 (blue) staining at 24 h after retinal detachment. The graphs show mean±S.E.M. Scale bar, 50 *μ*m

In contrast to the pro-death effects of mFasL, sFasL is reported to have pro-survival cell effects.^17, 18^ We previously demonstrated that the intravitreal injection of recombinant sFasL prevented TNFα-triggered RGC death,^22^ suggesting that sFasL might antagonize the activity of mFasL. In the current study, we injected recombinant murine sFasL (10 ng/1 *μ*l) or control phosphate-buffered saline (PBS) into the subretinal space when creating retinal detachment in B6129 ΔCS mice. The eyes treated with sFasL showed significantly reduced photoreceptor cell death as compared with control eyes (CTR: 1554±288 cells/mm^2^ and sFasL: 767±123 cells/mm^2^; *P*=0.045; Figures 5a and b). This indicates that sFasL antagonizes the mFasL activity in detached retinas and prevents photoreceptor cell death.

## Discussion

This study demonstrated that Fas/FasL-triggered cell death accounts for a significant amount of photoreceptor cell loss following retinal detachment, but not all cell death is due to Fas/FasL, as the complete absence of FasL in KO mice did not completely abolish photoreceptor cell death. In addition, we demonstrated that the function of FasL was dependent upon the form of FasL that was expressed. Membrane-bound FasL triggered photoreceptor cell death, while soluble FasL exhibited neuroprotective effects, indicating that enzyme-mediated cleavage of FasL determines, in part, the extent of vision loss following retinal detachment. Moreover, it also indicates that treatment with sFasL could significantly reduce photoreceptor cell loss in patients with detached retinas. There have been several previous reports examining the function of Fas signaling in photoreceptor cell death after retinal detachment using *Fas* or *FasL* mutant mice.^6, 20^ Hisatomi *et al.*^20^ reported that neither *Fas* deficiency (lpr/lpr) nor *FasL* deficiency (gld/gld) offered protection against photoreceptor cell death after retinal detachment. In contrast, Zacks *et al.*^6^ reported that *LPR* mice (lacking a functional Fas receptor) showed significantly reduced photoreceptor cell death after retinal detachment as compared with control mice. They also demonstrated that detachment-induced photoreceptor cell death was rescued by subretinal injection of Fas-neutralizing antibody, small interfering RNA against the Fas-receptor transcript (siFAS),^6^ or a small peptide inhibitor (Met12).^7^ Moreover, Zacks *et al.*^6^ indicated that the LPR mice used by Hisatomi *et al.*^20^ were bred on a C3H background, which carries the retinal degeneration 1 (*rd1*) allele.^6, 30^ Therefore, the use of these mice with an *rd1* mutation by Hisatomi *et al.* most likely confounded their results because the *rd1* retinal degeneration is driven predominantly by apoptosis-inducing factor and caspase 12.^31^ Moreover, the GLD and LPR mouse strains used by Zack *et al.*^6^ did not completely block Fas/FasL signaling since these mice have point mutations that allow residual activation of the Fas/FasL pathway, as reported by Karray *et al.*^21^ Unfortunately, FasL-overexpressing mice are not available, and we could not compare the photoreceptor cell death after retinal detachment between ΔCS and FasL-overexpressing mice, which remains a limitation of our study.

Using B6129 mice that are resistant to retinal detachment-induced photoreceptor cell death, we demonstrated that blocking enzymatic cleavage of FasL in B6129 ΔCS mice significantly increased the loss of photoreceptors following the retinal detachment. ΔCS mice have an exchange mutation in the FasL metalloproteinase binding site that prevents cleavage of mFasL to produce sFasL.^22^ Conversely, in WT mice, metalloproteinases cleave mFasL to produce sFasL, which reduces the Fas death signaling.^13, 17, 22^ This suggests that cleavage of FasL to produce sFasL is a protective mechanism to prevent photoreceptor cell death from Fas signaling after retinal detachment. Moreover, we previously reported using ΔCS mice that mFasL triggers retinal ganglion cell death in two different models of glaucoma.^22^ Taken together, these results implicate FasL cleavage as a potential mechanism for limiting the neurotoxic activity of mFasL in the eye.

The modified retinal detachment model we used in this study, in which the height of the detachment was increased, consistently showed the peak of photoreceptor cell death on day 1,^32, 33^ whereas previous studies (including those from our own group) that used a more shallow detachment reported that the peak of photoreceptor death was on day 3.^20, 34, 35^ These results are consistent with reports that photoreceptor cell death increases with the increasing height of retinal detachment.^36, 37^ Photoreceptor cell damage in higher detachments may be more extensive compared with shallow detachments because of reduced diffusion of oxygen and essential nutrients from the choriocapillaris.^38, 39^ Using our modified retinal detachment model, we create a detachment that is extremely bullous and persistent in 60% of the fundus, which appears to accelerate photoreceptor cell death in the detached retina more than other models.

In this study, we used two strains of mice that we previously demonstrated as susceptible (BALB/c) and resistant (B6129) to retinal detachment-induced photoreceptor death. When FasL was knocked out in susceptible BALB/c mice, there was a significant reduction in photoreceptor death. In contrast, when enzymatic cleavage was blocked in resistant B6129 ΔCS mice, there was a significant increase in photoreceptor death. Interestingly, TEM analysis revealed a reduction in both apoptotic and necrotic photoreceptor death in BALB/c *FasL*−/− mice. However, blocking the cleavage of FasL in B6129 ΔCS mice increased apoptotic photoreceptor death, but had no effect on necrotic cell death. These results suggest that both caspase-mediated apoptosis signaling and RIP-mediated necrotic signaling are attenuated in the absence of FasL. However, we speculate that increased mFasL in ΔCS mice might preferentially trigger the apoptosis signaling pathway over the necrotic signaling pathway.

Macrophages infiltrate into the subretinal space after a retinal detachment.^11, 24, 40, 41, 42, 43^ Hisatomi *et al.*^40^ reported that macrophages infiltrate from choroidal vessels into the subretinal space beyond the RPE layer. The RPE constitutively express FasL, which functions as an immune-privileged tissue ‘barrier' that induces apoptosis of infiltrating Fas+ cells, thereby blocking cell infiltration.^44, 45^ Moreover, it has also been reported that macrophages express Fas and readily undergo apoptosis when cultured *in vitro* with anti-Fas antibodies.^46, 47^ Using Ly6c, a maker expressed on bone-marrow derived monocytes, we recently demonstrated a significant increase in *Ly6c* mRNA expression in the subretinal space on day 1 as compared with day 7 following retinal detachment.^28^ Taken together, these data predict that FasL expressed on RPE will inhibit macrophage infiltration into the subretinal space 24 h after retinal detachment. Our data are consistent with this prediction, since in the absence of FasL in BALB/c *FasL*−/− mice we observed a significant increase in the infiltration of CD11b-positive cells into the subretinal space following retinal detachment, even in the presence of reduced expression of the chemotactic factor MCP-1. Moreover, blocking the cleavage of FasL in B6129 ΔCS mice, which increases the expression of pro-apoptotic mFasL, resulted in a significant reduction in the infiltration of CD11b-positive cells into the subretinal space, even in the presence of elevated expression of MCP-1. Together, these data support the hypothesis that the pro-apoptotic mFasL expressed on the RPE inhibits macrophage infiltration into the subretinal space following retinal detachment. However, it is possible that CD11b-positive cells that migrated into the subretinal space might not be macrophages, but rather other immune cells (e.g., natural killer cells). FasL can affect the function of CD11b-positive cells and cause the discrepancy between MCP-1 expression and migration of CD11b-positive cells after retinal detachment, which remains to be elucidated.

It has been shown that classically activated (M1) and alternatively activated (M2) macrophages have opposite roles in inflammation and wound healing.^48, 49, 50^ M1 macrophages generate high levels of pro-inflammatory cytokines and produce reactive nitrogen and oxygen intermediates. In contrast, M2 macrophages have immunoregulatory functions, promote tissue remodeling, express high levels of scavenger receptors, and display efficient phagocytic activity. In the current study, we demonstrated that the subtype of infiltrating macrophages changes with time after retinal detachment: M1 macrophages are the predominant cell type during the acute phase (day 1) and M2 macrophages are the predominant cell type during the late phase (day 7). In addition, the CD11b-positive cell density was higher in the late phase as compared with the acute phase, which corresponded with a higher frequency of TUNEL-positive cells in the acute phase. The switch from M1 to M2 subtype may be a partial reason for the endogenous neuroprotection seen after retinal detachment that leads to the observed decline in the rate of cell death over time. However, neither the mouse strain nor FasL had any effect on the timing or subtype of infiltrating macrophages during retinal detachment.

Murine sFasL is reported to be non-apoptotic and anti-inflammatory, and can even act as an antagonist by blocking mFasL engagement with the Fas receptor.^17, 18^ We previously demonstrated the neuroprotective effects of sFasL in a TNFα-triggered model of retinal ganglion cell neurotoxicity.^22^ In the current study, we simultaneously injected recombinant sFasL into the subretinal space when the retinal detachment was created, which attenuated the subsequent photoreceptor cell death. These data suggest that sFasL might antagonize the activity of mFasL and prevent photoreceptor cell death following retinal detachment.

In conclusion, using *FasL*−/− and ΔCS mice, we demonstrated that the different forms of FasL display opposite functions following the retinal detachment: mFasL increased loss of photoreceptors, whereas sFasL inhibited cell death. In addition, mFasL expressed on the RPE may limit inflammatory M1 cell infiltration during the acute phase following retinal detachment, independent of the presence of pro-inflammatory cytokines and chemokines. This blockade was reduced during the later phase of retinal detachment allowing increased numbers of M2 macrophages into the wounded retina. Understanding the mechanisms of FasL-induced photoreceptor cell death and endogenous neuroprotection will provide new therapeutic targets for preventing loss of photoreceptors in retinal diseases affected by separation of photoreceptors from RPE, such as rhegmatogenous retinal detachment.

## Materials and Methods

### Animals

All animal experiments followed the guidelines of the ARVO Statement for the Use of Animals in Ophthalmic and Vision Research and were approved by the Animal Care Committee of the Massachusetts Eye and Ear Infirmary. *FasL*−/− mice on the BALB/c background were provided by Ann Marshak-Rothstein (Supplementary Figure S1).^22^ BALB/c mice were purchased from Charles River Laboratories (Wilmington, MA, USA) as control WT mice for *FasL*−/− mice. ΔCS mice on the B6129 background were provided by Ann Marshak-Rothstein (Supplementary Figure S1).^22^ B6129SF2 mice were purchased from Jackson Laboratories (Bar Harbor, ME, USA) as control WT mice for ΔCS mice. Mice were fed standard laboratory chow and allowed free access to water in an air-conditioned room with a 12-h light/12-h dark cycle. All mice were used at postnatal 8±1 weeks.

### Creation of retinal detachment

We modified a previously reported method for creating experimental retinal detachments and achieved bullous and persistent retinal detachments.^51^ Briefly, mice were anesthetized with an intraperitoneal injection of a mixture of 60 mg/kg ketamine and 6 mg/kg xylazine, and pupils were dilated with topical phenylephrine (5%) and tropicamide (0.5%). The temporal conjunctiva at the posterior limbus was incised and detached from the sclera. A 30-gauge needle (BD, Franklin Lakes, NJ, USA) was used with the bevel pointed up to create a sclerotomy 1 mm posterior to the limbus. A scleral tunnel was created followed by scleral penetration into the choroid, which makes a self-sealing scleral wound. A corneal puncture was made with a 30-gauge needle to lower intraocular pressure. Then, a 34-gauge needle connected to a 10-*μ*l syringe (NanoFil 10 *μ*l syringe; WPI, Sarasota, FL, USA) with the bevel pointed down was inserted into the subretinal space and 4 *μ*l of 1% sodium hyaluronate (Provisc; Alcon, Fort Worth, TX, USA) was injected gently to detach the neurosensory retina from the underlying RPE. Approximately 60% of the temporal-nasal neurosensory retina was detached. For evaluation of the efficacy of sFasL to prevent photoreceptor cell death after retinal detachment, recombinant murine sFasL (10 ng/1 *μ*l; R&D Systems, Inc., Minneapolis, MN, USA) or control PBS was injected into the subretinal space followed by injection of 4 *μ*l of 1% sodium hyaluronate. Finally, cyanoacrylate surgical glue (WebglueTM; Patterson Veterinary, Devens, MA, USA) was put on the scleral wound, and the conjunctiva was reattached to the original position. Any eyes with subretinal hemorrhage were excluded from the study.

### TdT-dUTP terminal nick-end labeling

Following retinal detachments, eyes were enucleated at multiple time points and embedded in OCT compound (Tissue Tek; Sakura Finetec, Torrance, CA, USA). Serial sections of the eyes in the sagittal plane were cut at 10 *μ*m thickness on a cryostat (CM1850; Leica, Heidelberg, Nussloch, Germany) at −20 °C and prepared for staining. TUNEL assay was performed according to the manufacturer's protocol (ApoTag Fluorescein *In Situ* Apoptosis Detection Kit; Millipore, Billerica, MA, USA). Finally, sections were counterstained with TO-PRO-3. The number of TUNEL-positive cells was counted in the ONL, which contains the photoreceptors. The area of ONL was also measured by the Image J software (developed by Wayne Rasband, National Institutes of Health, Bethesda, MD, USA; available at http://rsb.info.nih.gov/ij/index.html), and then TUNEL-positive cell density in ONL was calculated. A preliminary experiment revealed that the center of the retinal detachment had less variability of TUNEL-positive cell density (data not shown). Thus, TUNEL-positive cell density was evaluated using sections around 1000 *μ*m from the injection site.^23, 32^ Photographs were taken by confocal microscopy using a HCX APOL 40x lens (Leica, Allendale, NJ, USA).

### Evaluation of ONL/INL ratio

The ONL and INL areas of the detached retina were measured by the Image J software, and the ONL/INL ratio was calculated. Areas of abnormal retinal morphology were excluded so that uniform unbiased measurements can be obtained.

### Transmission electron microscopy

TEM was performed as previously described.^52^ More than 200 photoreceptors per eye were photographed and subjected to quantification of cell death modes in a masked manner. Photoreceptors showing cellular shrinkage and nuclear condensation were defined as apoptotic cells, whereas photoreceptors associated with cellular and organelle swelling and discontinuities in plasma and nuclear membrane were defined as necrotic cells. Electron-dense granular materials were labeled simply as end-stage cell death/unclassified, because these materials are reported to occur subsequently to both apoptotic and necrotic cell death.

### ELISA for MCP-1 and IL-6

The levels of MCP-1 and IL-6 were determined with mouse MCP-1 and IL-6 ELISA kits (R&D Systems, Inc.), according to the manufacturer's protocol.

### Immunohistochemistry

Sections were fixed in acetone for 5 min, blocked in 2% skim milk for 20 min, and incubated with rat anti-CD11b antibody (1 : 50; BD Biosciences, San Jose, CA, USA) at 4 °C overnight. Alexa Fluor 488-conjugated goat anti-rat IgG was used as a secondary antibody and incubated at room temperature for 30 min. Finally, sections were counterstained with TO-PRO-3.

### Laser capture microdissection

LCM was performed with Leica LMD 7000 (Leica Microsystems, Buffalo Grove, IL, USA). After enucleation, eyes were frozen in OCT compound and cut into 40 *μ*m sections on RNase-free polyethylene naphthalate membrane slides (Leica Microsystems). The sections were fixed and dehydrated with 75, 95, and 100% ethanol. The tissues from subretinal space were microdissected and collected into 0.5-ml tubes containing RNAlater (Life Technologies, Grand Island, NY, USA).

### Measurement of mRNA expression by qPCR

The subretinal tissue dissected by LCM was used for qPCR. Total RNA was harvested using the RNeasy Plus Micro Kit (Qiagen, Valencia, CA, USA). cDNA was generated with Oligo-dT primer (Invitrogen, Camarillo, CA, USA) and Superscript III (Invitrogen) according to the manufacturer's instructions. Quantitative PCR was carried out using Ccr2 (Mm99999051_gH), Ly6c (Mm03009946_m1), Cx3cr1 (Mm02620111_s1), Il6 (Mm00446190_m1), and Rn18s (Mm03928990_g1) TaqMan gene expression assay (Applied Biosystems, Foster City, CA, USA).

### Statistical analysis

The results are expressed as the mean±S.E. Statistical differences between two groups were analyzed by Mann–Whitney *U* test. Multiple-group comparison was performed by two-way ANOVA followed by Bonferroni's post-test. The significance level was set at *P*<0.05 (* in figures), *P*<0.01 (** in figures), *P*<0.001 (*** in figures). Statistical analysis and graphing were performed using Prism Ver.5 (GraphPad Software, La Jolla, CA, USA).

## Abbreviations

FasL: Fas ligand
mFasL: membrane-bound FasL
sFasL: soluble FasL
RPE: retinal pigment epithelium
FADD: Fas-associated death domain
CAD: caspase-activated DNAse
RIP: receptor interacting protein
DISC: death-inducing signaling complex
PBS: phosphate-buffered saline
TUNEL: TdT-dUTP terminal nick-end labeling
ONL: outer nuclear layer
INL: inner nuclear layer
TEM: transmission electron microscopy
MCP-1: monocyte chemoattractant protein 1
IL-6: interleukin 6
LCM: laser capture microdissection
qPCR: quantitative real-time PCR
WT: wild type
CTR: control
siFAS: small interfering RNA against the Fas-receptor transcript
