# Supplementary figures and images for: Membrane-bound and soluble Fas ligands have opposite functions in photoreceptor cell death following separation from the retinal pigment epithelium
Source: Cell Death Dis. 2015 Nov 19;6(11):e1986–. doi: 10.1038/cddis.2015.334 (PMC4670938; doi:10.1038/cddis.2015.334)

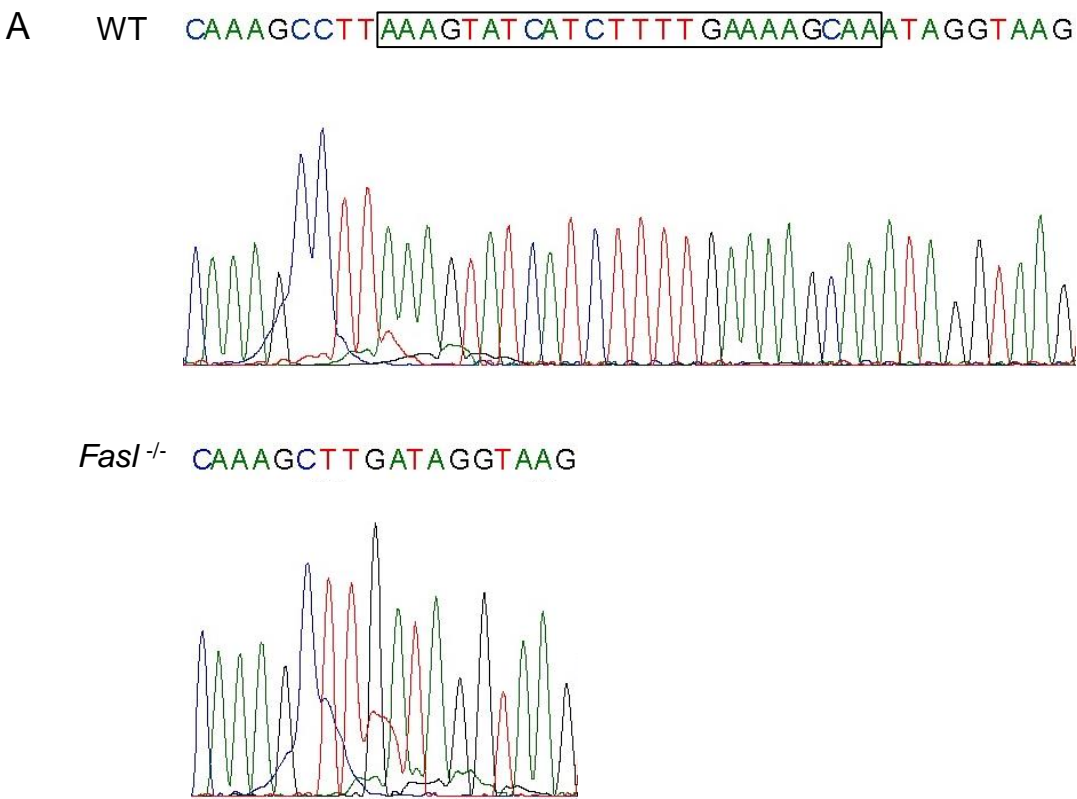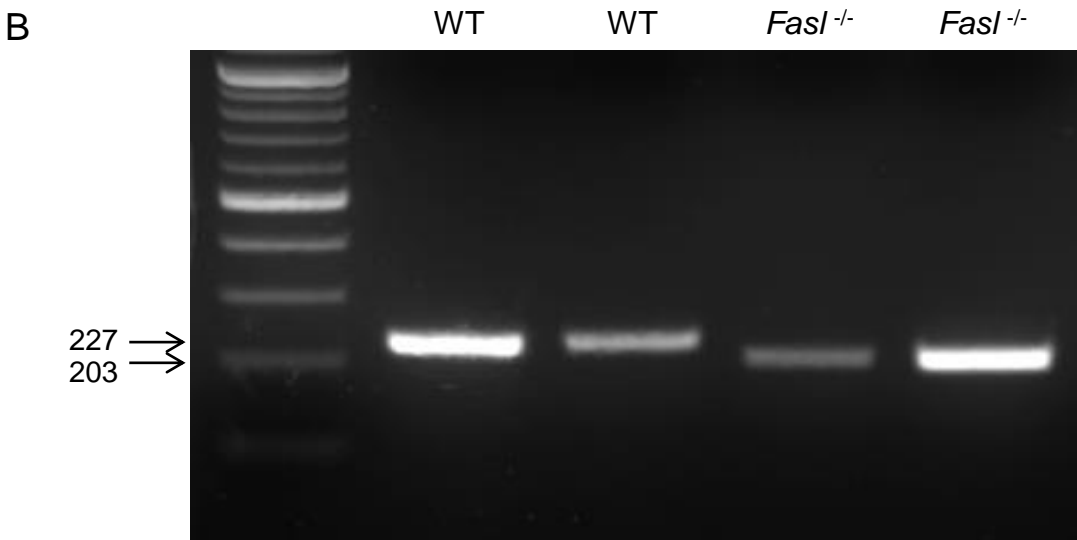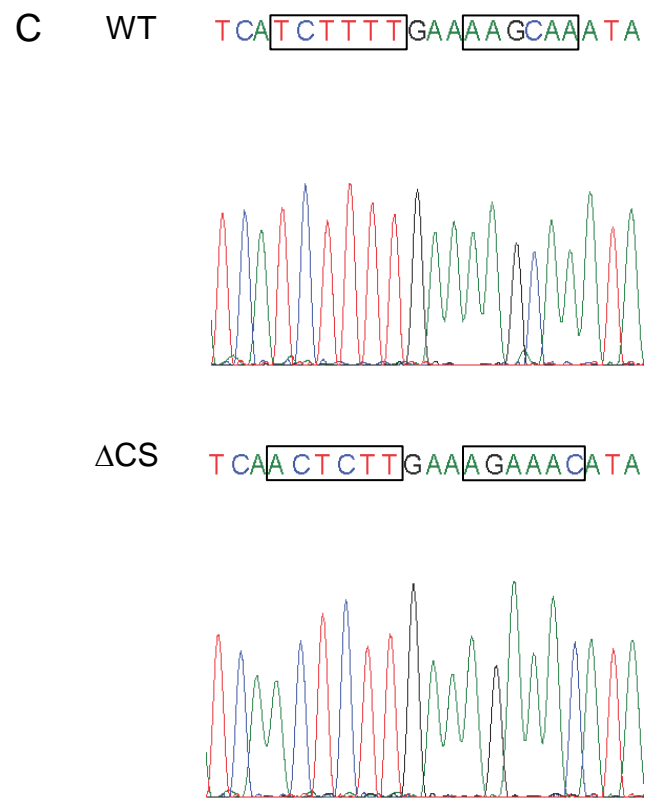

Supplement: Supplementary Figure 1 [file cddis2015334x1.pdf]

A

WT

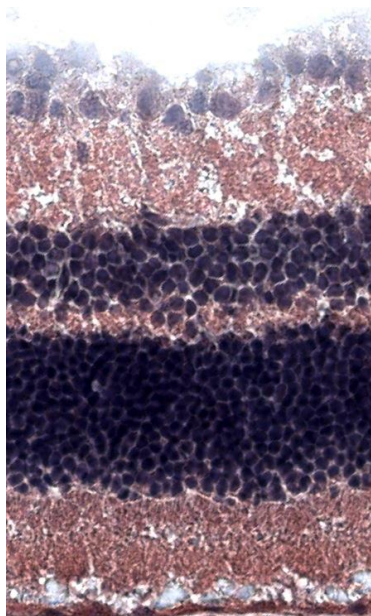

*FasI*<sup>-/-</sup>

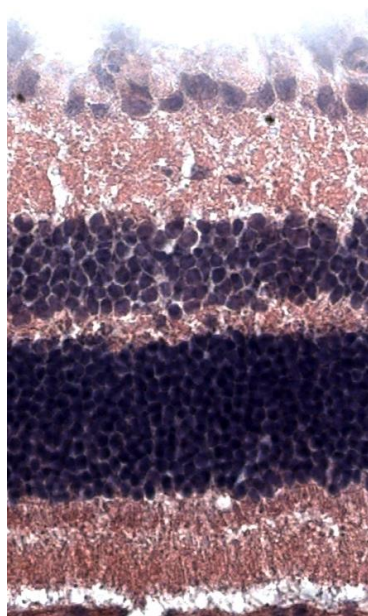

B

WT

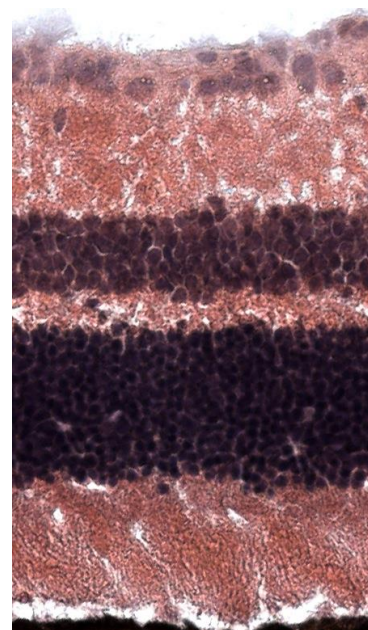

$\Delta$ CS

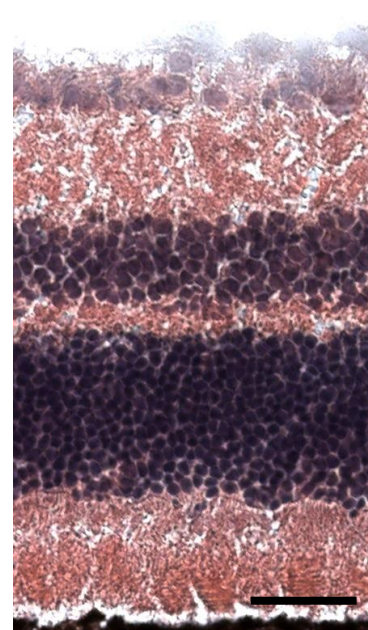

GCL

INL

ONL

RPE

Supplement: Supplementary Figure 2 [file cddis2015334x2.pdf]

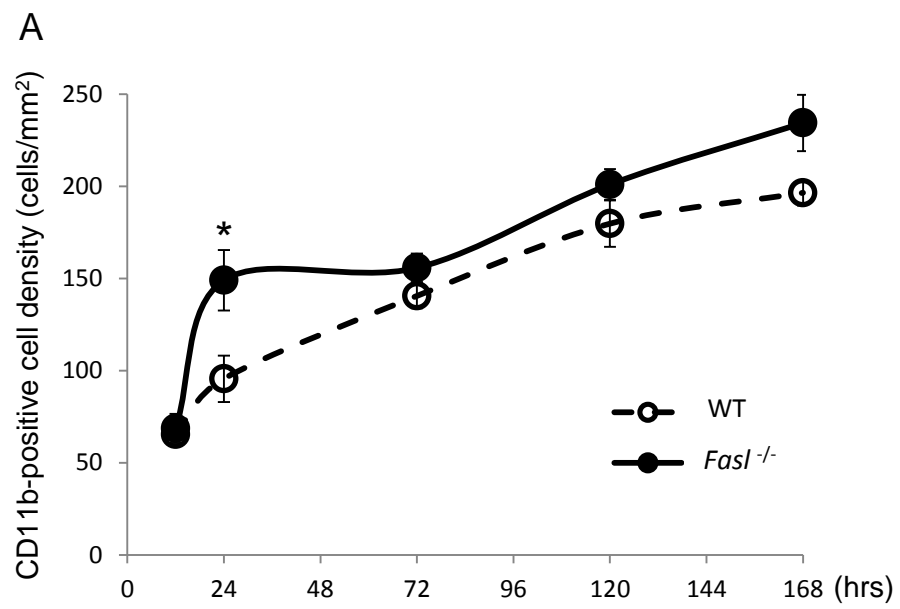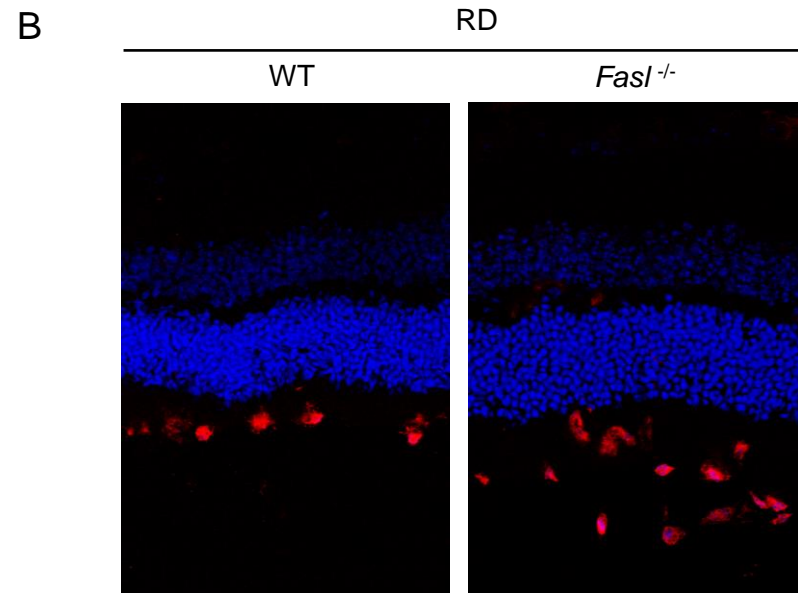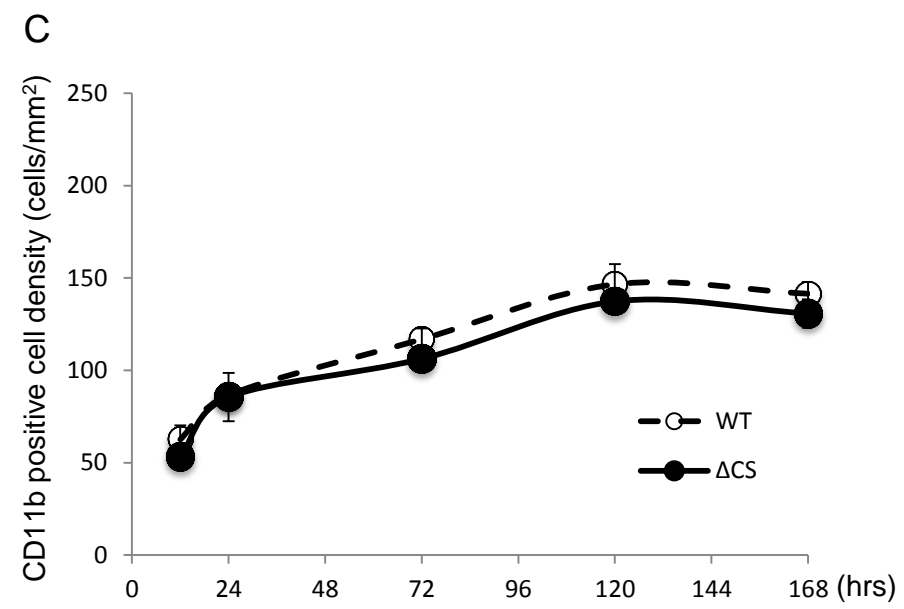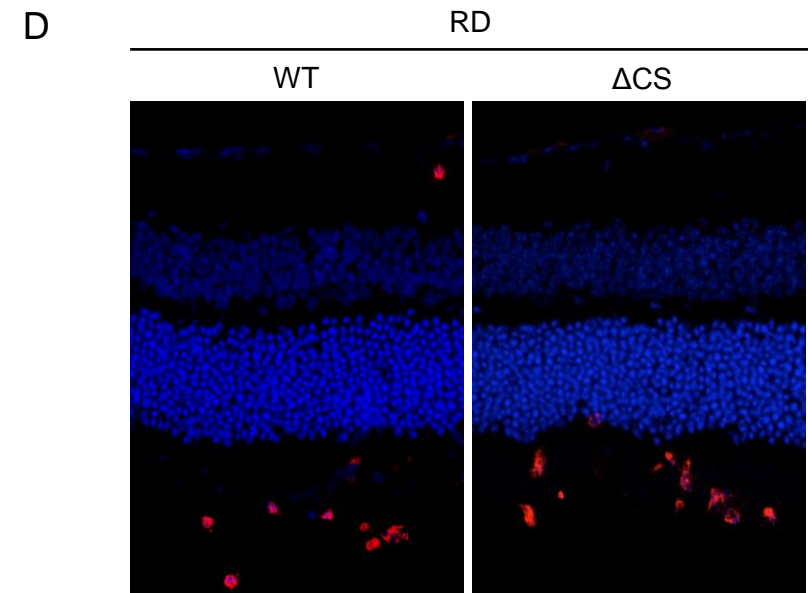

Supplement: Supplementary Figure 3 [file cddis2015334x3.pdf]
