# Supplementary material for: Membrane-bound and soluble Fas ligands have opposite functions in photoreceptor cell death following separation from the retinal pigment epithelium
Source: Cell Death Dis. 2015 Nov 19;6(11):e1986–. doi: 10.1038/cddis.2015.334 (PMC4670938; doi:10.1038/cddis.2015.334)
Supplement: Supplementary Figure Legends [file cddis2015334x4.doc]

**Figure S1** Mutations producing FasL knockout and ΔCS mice.

(**A**) DNA sequencing analysis shows the deletion of 8 nucleotides from the *FasL* gene sequence. This mutation results in a splicing error and frameshift mutation, thereby creating *FasL-/-* mice.^22^ (**B**) PCR was performed to amplify the region of *FasL* DNA. The results demonstrate that WT mice express a 227 bp fragment, while *FasL-/-* mice express a 203 bp fragment, which are consistent with the results of DNA sequencing analysis. (**C**) DNA sequencing analysis shows the exchange mutations in *FasL* gene sequence, which replaces the 4 residues bracketing the 2 potential cleavage sites in ΔCS strain. These exchange mutations eliminate the cleavage sites within the full-length protein and prevent the cleavage of FasL to produce the sFasL ^22^.

**Figure S2** Histology in untreated *FasL-/-* and ΔCS retinas.

The mutations in *FasL-/-* and ΔCS mice did not alter the retinal architecture as determined by histological examination. (**A**) H&E retinal cross-section from WT and *FasL-/-* mice. (**B**) H&E retinal cross-section from WT and ΔCS mice.

**Figure S3** Retinal detachment induced inflammatory cell infiltration is accelerated in *FasL-/-* mice.

(**A, B**) Time course of CD11b-positive cell density (*n*=6 each group and time point) (**A**) and CD11b (red) and TO-PRO-3 (blue) staining at 24 hours after retinal detachment (**B**) in BALB/c WT and BALB/c *FasL-/-* mice. Infiltration of CD11b-positive cells was significantly higher in BALB/c *FasL-/-* mice at 24 hours after retinal detachment (**P*<0.05). By 3 days after retinal detachment, the CD11b-positive cell density was equivalent in both BALB/c WT and BALB/c *FasL-/-* mice. (**C, D**) Time course of CD11b-positive cell density (*n*=6 each group and time point) (**C**) and CD11b (red) and TO-PRO-3 (blue) staining at 24 hours after retinal detachment (**D**) in B6129 WT and B6129 ΔCS mice. Infiltration of CD11b-positive cells was equivalent in both groups at any time points. The graphs show mean±S.E.M. Scale bar, 50 µm.
